# Supplementary material for: Comparison of the Effectiveness of Palonosetron and Ramosetron in Preventing Postoperative Nausea and Vomiting: Updated Systematic Review and Meta-Analysis with Trial Sequential Analysis
Source: J Pers Med. 2022 Dec 29;13(1):82. doi: 10.3390/jpm13010082 (PMC9866437; doi:10.3390/jpm13010082)
Supplement: Supplementary file 1 [file jpm-13-00082-s001.zip › jpm-2081251-supplementary.pdf]

Appendix

Search terms for MEDLINE

1. randomized controlled trial.pt
2. randomized controlled trial$.mp
3. controlled clinical trial.pt
4. controlled clinical trial$.mp
5. random allocation.mp
6. exp double-blind method/
7. double-blind.mp
8. exp single-blind method/
9. single-blind.mp
10. or/1-9
11. clinical trial.pt
12. clinical trial$.mp
13. exp clinical trial/
14. (clin$ adj25 trial$).mp
15. ((singl$ or doubl$ or tripl$ or trebl$) adj25 (blind$ or mask$)).mp
16. random$.mp
17. exp research design/
18. research design.mp
19. or/11-18
20. 10 or 19
21. Case report.tw.
22. Letter.pt.
23. Historical article.pt.
24. Review.pt.
25. or/21-24
26. 20 not 25
27. Palonosetron.mp.
28. Aloxi
29. Or/27-28
30. Ramosetron.mp.
31. **Ibset.mp.**

32. **Iribo.mp.**
33. **Nozia.mp.**
34. **Nasea.mp.**
35. Or/30-34
36. 29 and 35
37. 26 and 36

Search terms for Embase

1. randomized controlled trial$.mp.
2. 'controlled clinical trial (topic)'/exp
3. controlled AND clinical AND trials
4. controlled clinical trial$.mp.
5. 'randomization'/exp
6. 'random allocation'/exp
7. random allocation.mp.
8. double-blind.mp.
9. single-blind.mp.
10. #1 OR #2 OR #3 OR #4 OR #5 OR #6 OR #7 OR #8 OR #9
11. 'clinical trial (topic)'/exp
12. clinical AND trial$.mp.
13. random$.mp.
14. rct
15. #11 OR #12 OR #13 OR #14
16. #10 OR #15
17. 'case study'/exp
18. 'case report'/exp
19. 'abstract report'/exp
20. 'letter'/exp
21. #17 OR #18 OR #19 OR #20
22. #16 NOT #21
23. Palonosetron.mp.
24. Aloxi

25. Or/27-28
26. Ramosetron.mp.
27. **Ibset.mp.**
28. **Iribo.mp.**
29. **Nozia.mp.**
30. **Nasea.mp.**
31. Or/30-34
32. 29 and 35
33. 26 and 36

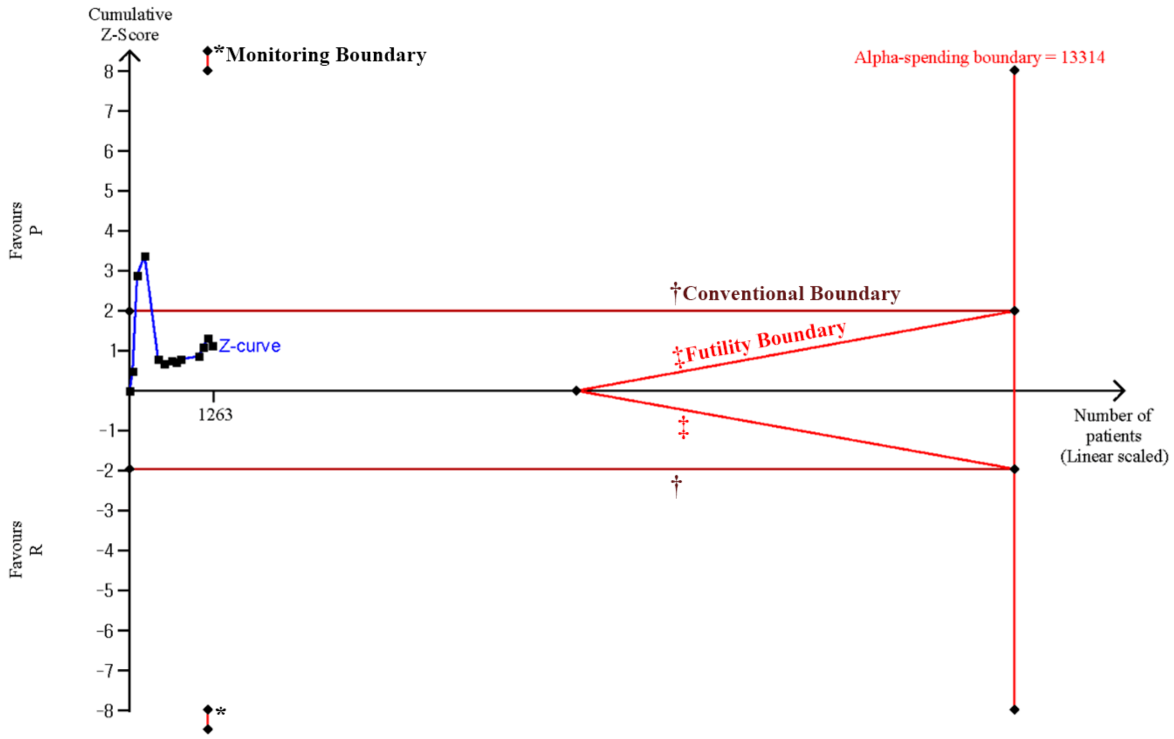

Supplementary Figure 1A. **The trial sequential analysis (TSA) for the studies comparing the effect of palonosetron to that of ramosetron on early PON.** The cumulative Z curve did not cross the conventional test boundary and stayed within the statistically insignificant zone. TSA indicated that only 1,263 of 13,314 patients of the required information size (RIS) accumulated. Uppermost and lowermost curves represent trial sequential monitoring boundary lines (*) for benefit and harm respectively. Two horizontal lines represents the conventional boundaries (†) for statistical significance. Triangular lines on the right side reflects the futility boundaries (‡). The number on the x-axis indicates RIS.

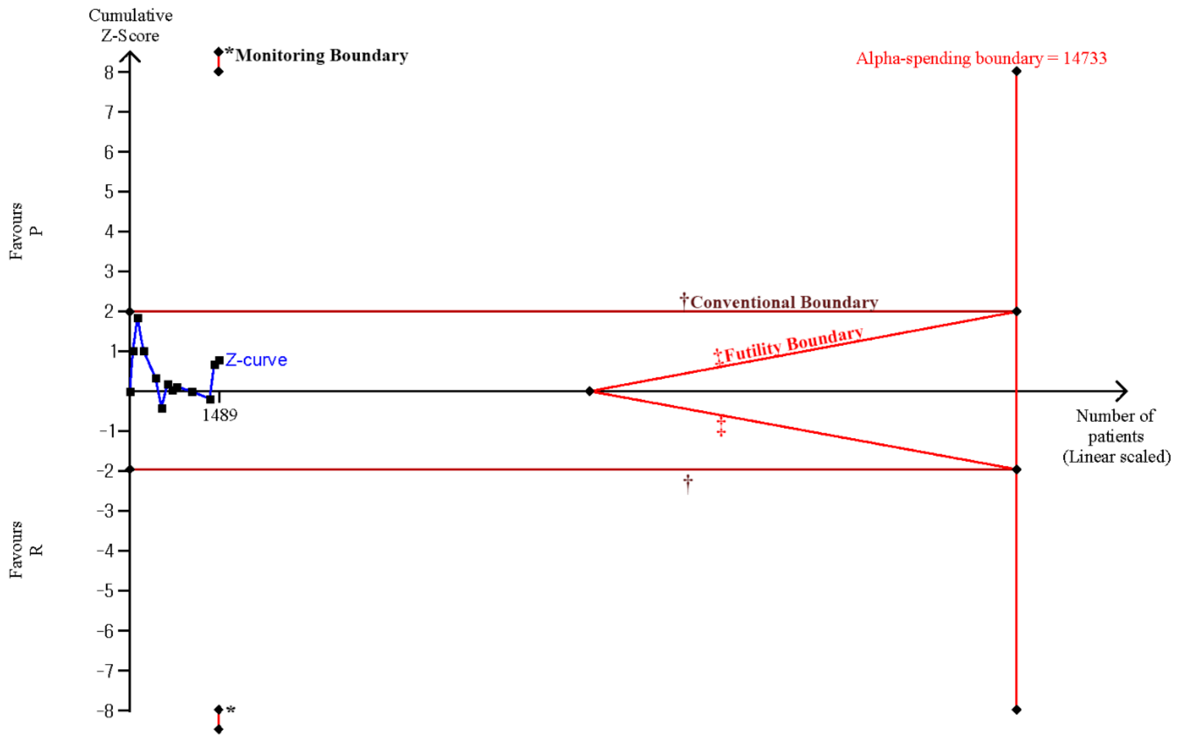

Supplementary Figure 1B. **The trial sequential analysis for the studies comparing the effect of palonosetron to that of ramosetron on late PON**. The cumulative Z curve did not cross the conventional test boundary and stayed within the statistically insignificant zone. TSA indicated that only 1,489 of 14,733 patients of the required information size (RIS) accumulated. Uppermost and lowermost curves represent trial sequential monitoring boundary lines (*) for benefit and harm respectively. Two horizontal lines represents the conventional boundaries (†) for statistical significance. Triangular lines on the right side reflects the futility boundaries (‡). The number on the x-axis indicates RIS.

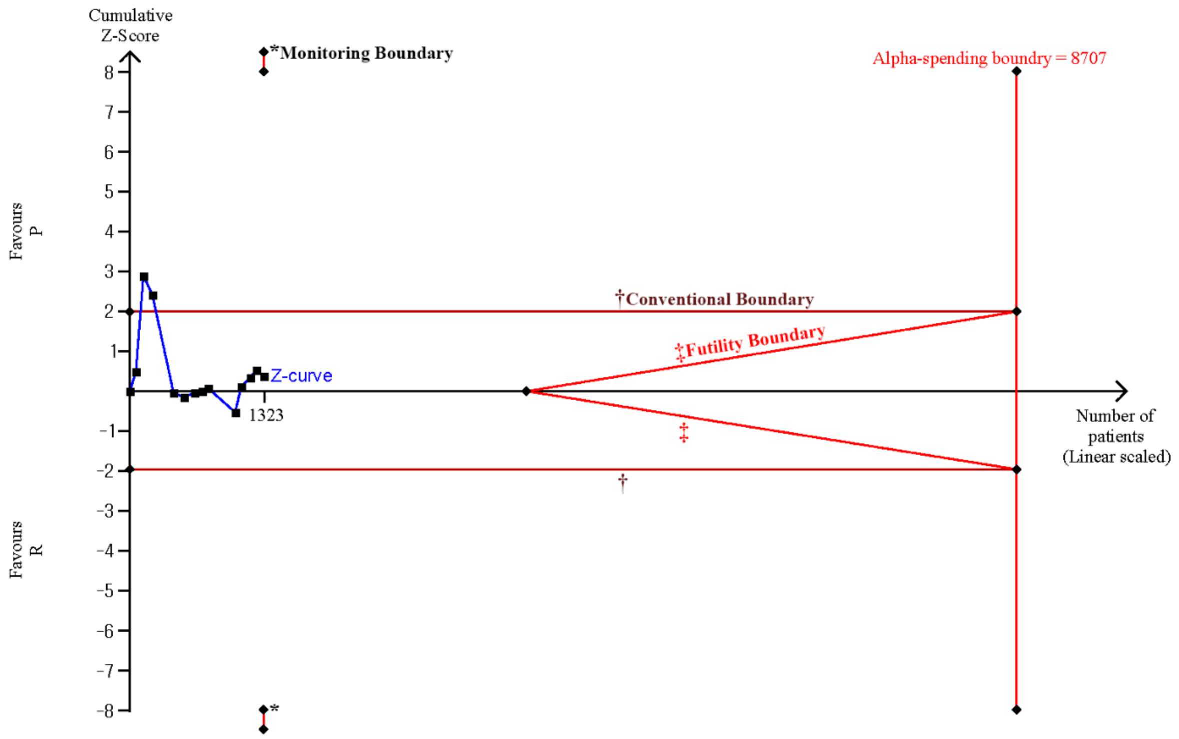

Supplementary Figure 1C. **The trial sequential analysis for the studies comparing the effect of palonosetron to that of ramosetron on overall PON.** The cumulative Z curve did not cross the conventional test boundary and stayed within the statistically insignificant zone. TSA indicated that only 1,323 of 8,707 patients of the required information size (RIS) accumulated. Uppermost and lowermost curves represent trial sequential monitoring boundary lines (*) for benefit and harm respectively. Two horizontal lines represents the conventional boundaries (†) for statistical significance. Triangular lines on the right side reflects the futility boundaries (‡). The number on the x-axis indicates RIS.

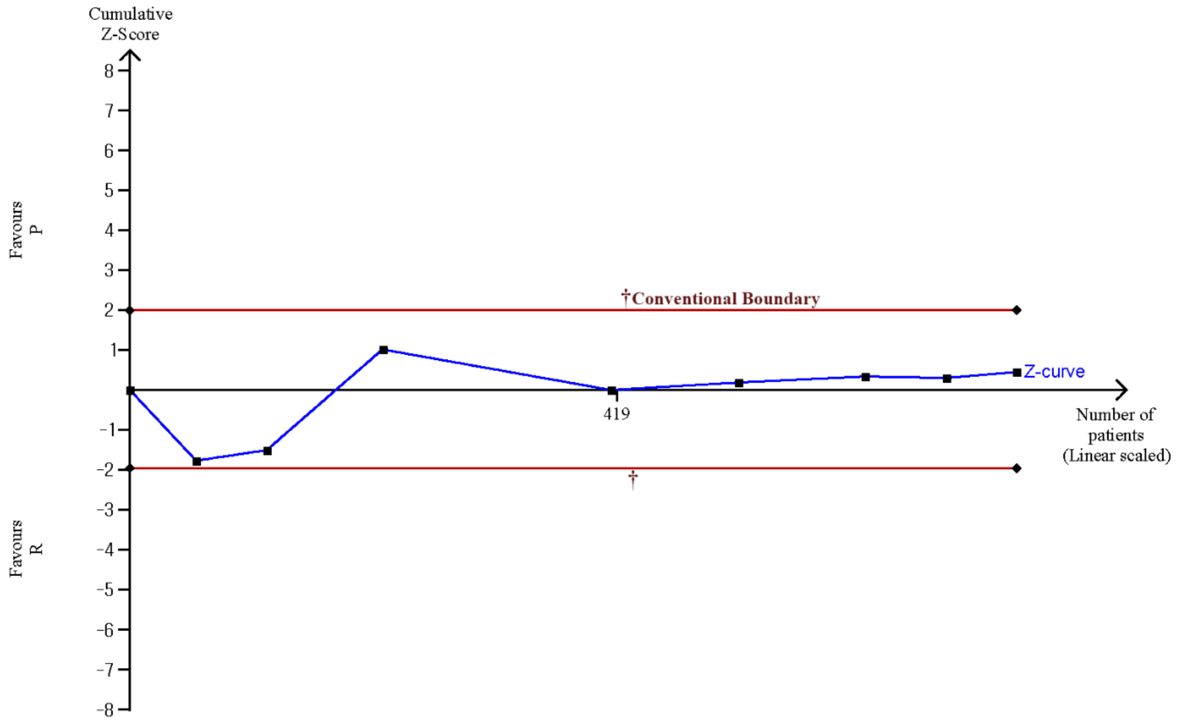

Supplementary Figure 2A. **The trial sequential analysis for the studies comparing the effect of palonosetron to that of ramosetron on early POV.** The cumulative Z curve did not cross the conventional test boundary and stayed within the statistically insignificant zone. TSA indicated that only 1,097 of 23,261 patients of the required information size (RIS) accumulated. Two horizontal lines represents the conventional boundaries (†) for statistical significance.

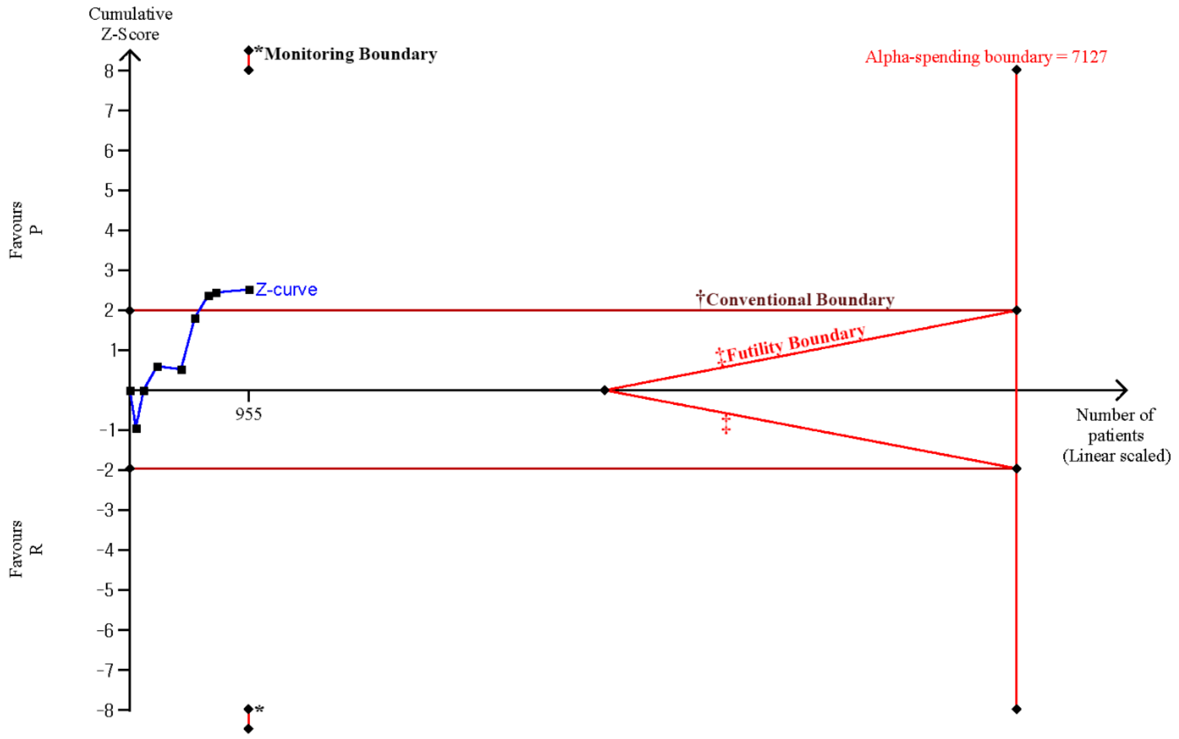

Supplementary Figure 2B. **The trial sequential analysis for the studies comparing the effect of palonosetron to that of ramosetron on late POV.** The cumulative Z curve crossed the conventional test boundary but did not cross the trial sequential monitoring boundary. TSA indicated that only 1,097 of 7,127 patients of the required information size (RIS) accumulated. Uppermost and lowermost curves represent trial sequential monitoring boundary lines (*) for benefit and harm respectively. Two horizontal lines represents the conventional boundaries (†) for statistical significance. Triangular lines on the right side reflects the futility boundaries (‡). The number on the x-axis indicates RIS.

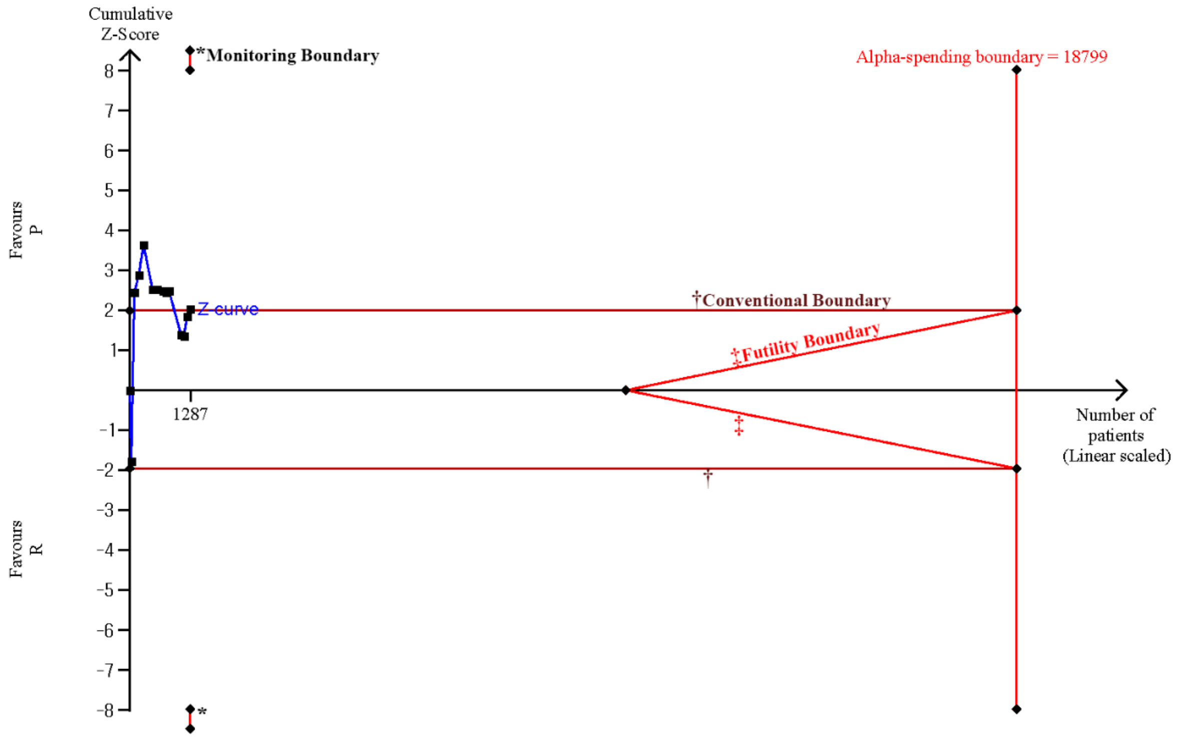

Supplementary Figure 2C. **The trial sequential analysis for the studies comparing the effect of palonosetron to that of ramosetron on overall POV**. The cumulative Z curve crossed the conventional test boundary but did not cross the trial sequential monitoring boundary. TSA indicated that only 1,359 of 18,799 patients of the required information size (RIS) accumulated. Uppermost and lowermost curves represent trial sequential monitoring boundary lines (*) for benefit and harm respectively. Two horizontal lines represents the conventional boundaries (†) for statistical significance. Triangular lines on the right side reflects the futility boundaries (‡). The number on the x-axis indicates RIS.

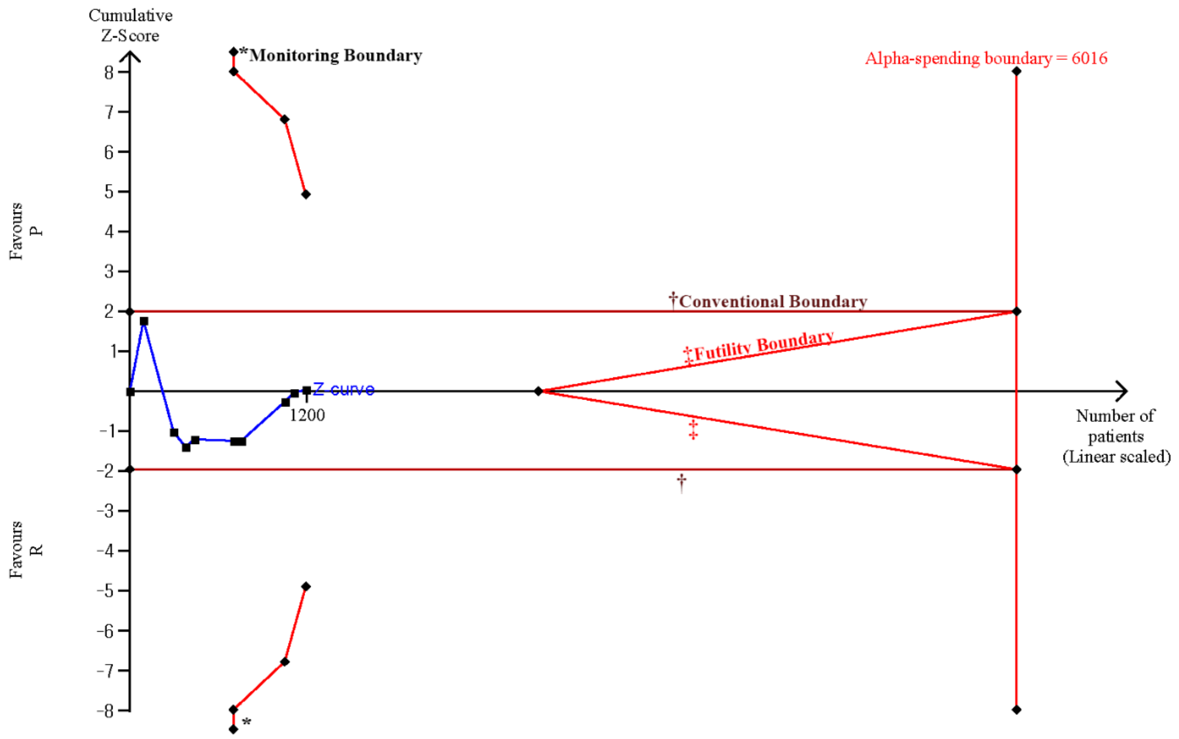

Supplementary Figure 3A. **The trial sequential analysis for the studies comparing the effect of palonosetron to that of ramosetron on early PONV.** The cumulative Z curve did not cross the conventional test boundary and stayed within the statistically insignificant zone. TSA indicated that only 1,200 of 6,016 patients of the required information size (RIS) accumulated. Uppermost and lowermost curves represent trial sequential monitoring boundary lines (*) for benefit and harm respectively. Two horizontal lines represents the conventional boundaries (†) for statistical significance. Triangular lines on the right side reflects the futility boundaries (‡). The number on the x-axis indicates RIS.

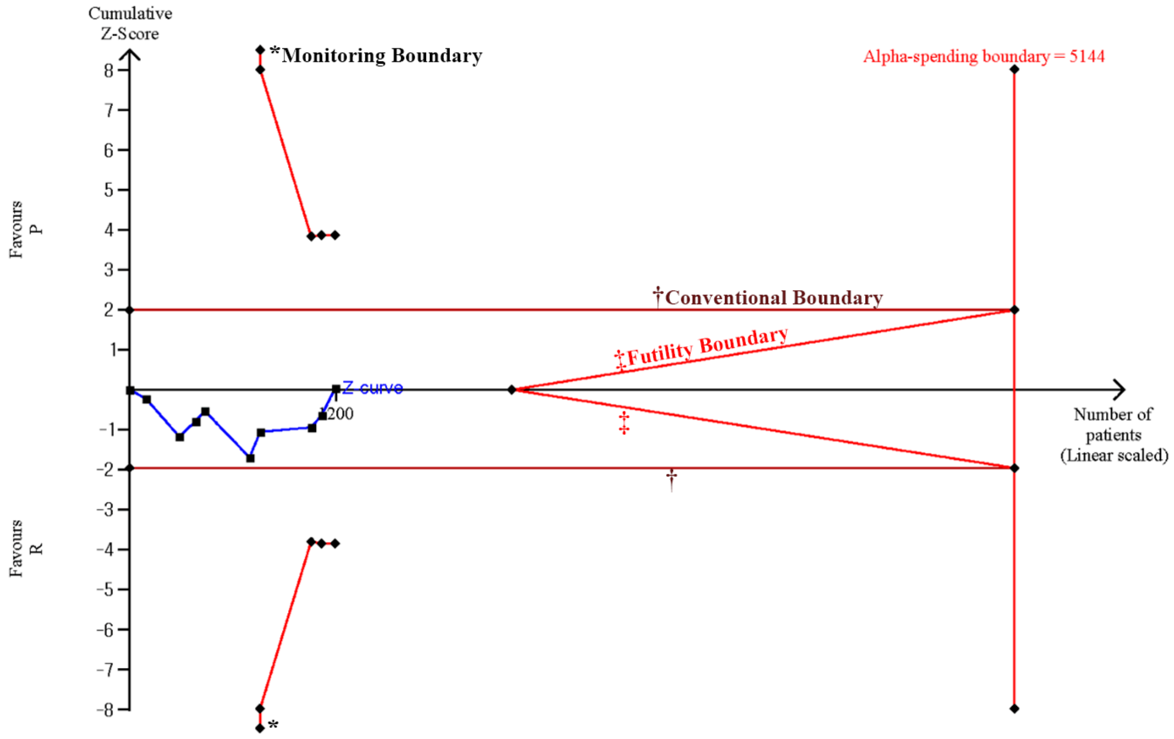

Supplementary Figure 3B. **The trial sequential analysis for the studies comparing the effect of palonosetron to that of ramosetron on late PONV**. The cumulative Z curve did not cross the conventional test boundary and stayed within the statistically insignificant zone. TSA indicated that only 1,200 of 5,144 patients of the required information size (RIS) accumulated. Uppermost and lowermost curves represent trial sequential monitoring boundary lines (*) for benefit and harm respectively. Two horizontal lines represents the conventional boundaries (†) for statistical significance. Triangular lines on the right side reflects the futility boundaries (‡). The number on the x-axis indicates RIS.

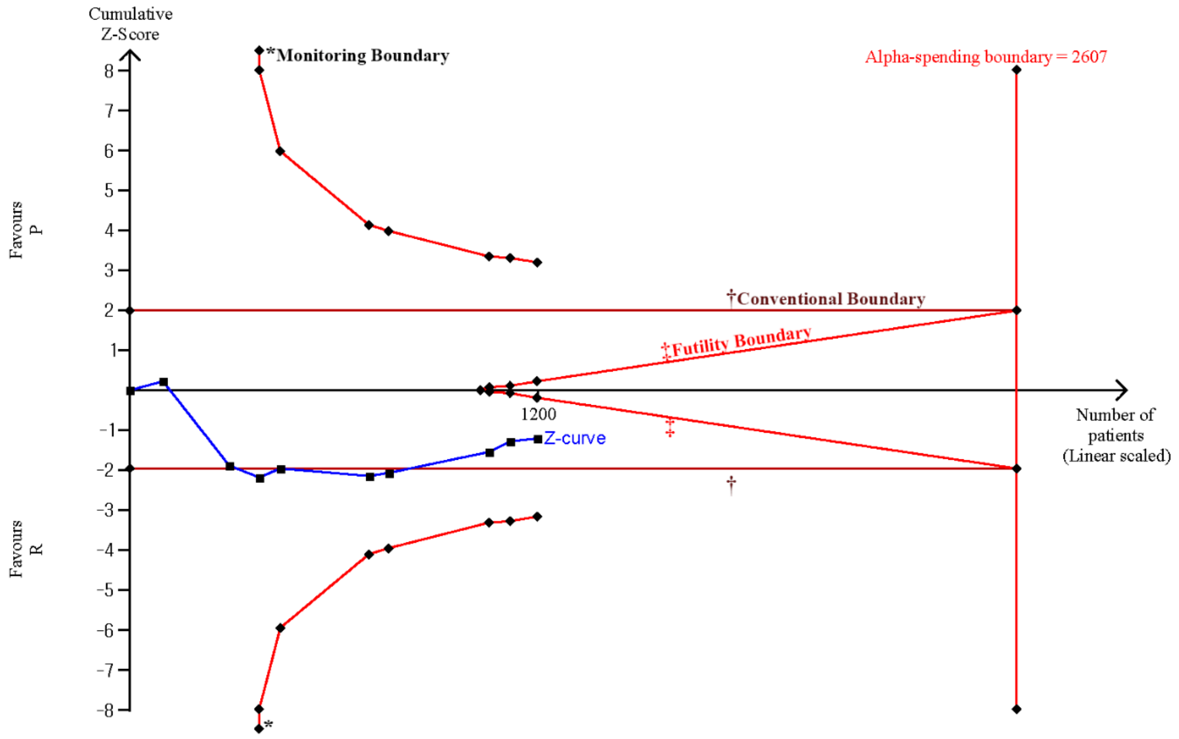

Supplementary Figure 3C. **The trial sequential analysis for the studies comparing the effect of palonosetron to that of ramosetron on overall PONV.** The cumulative Z curve did not cross the conventional test boundary and stayed within the statistically insignificant zone. TSA indicated that only 1,200 of 2,607 patients of the required information size (RIS) accumulated. Uppermost and lowermost curves represent trial sequential monitoring boundary lines (*) for benefit and harm respectively. Two horizontal lines represents the conventional boundaries (†) for statistical significance. Triangular lines on the right side reflects the futility boundaries (‡). The number on the x-axis indicates RIS.

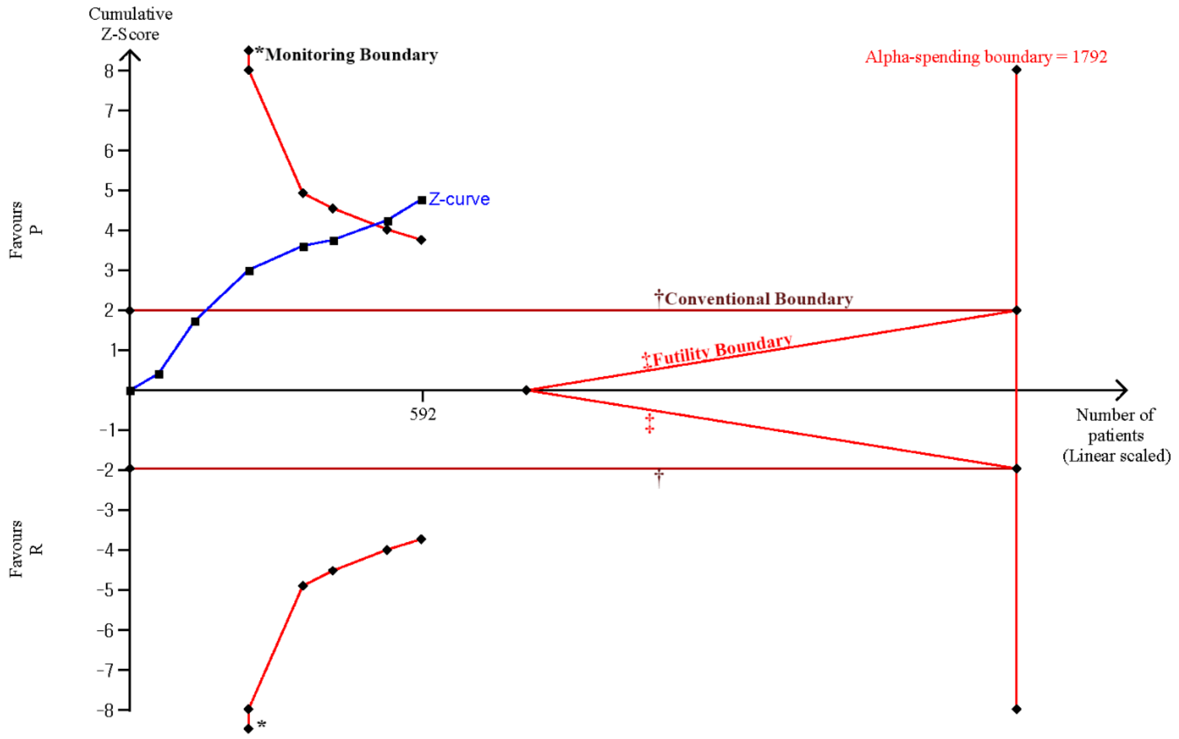

Supplementary Figure 4A. The trial sequential analysis for the studies comparing the effect of palonosetron to that of ramosetron on **retching**. The cumulative Z curve crossed both the conventional test boundary and the trial sequential monitoring boundary to reach area of benefit. TSA indicated that only 592 of 1,792 patients of the required information size (RIS) accumulated. Uppermost and lowermost curves represent trial sequential monitoring boundary lines (*) for benefit and harm respectively. Two horizontal lines represents the conventional boundaries (†) for statistical significance. Triangular lines on the right side reflects the futility boundaries (‡). The number on the x-axis indicates RIS.

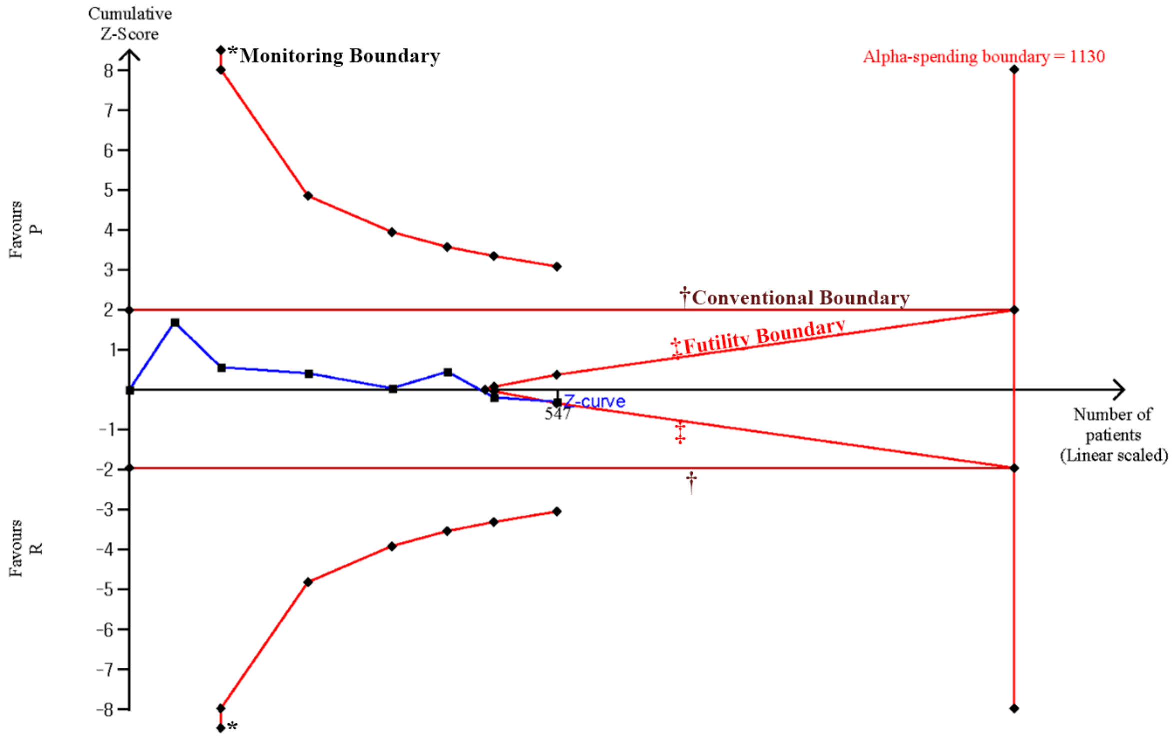

Supplementary Figure 4B. **The trial sequential analysis for the studies comparing the effect of palonosetron to that of ramosetron on complete response.** The cumulative Z curve crossed the futility boundary to conclude that the intervention has no effect. TSA indicated that only 547 of 1,130 patients of the required information size (RIS) accumulated. Uppermost and lowermost curves represent trial sequential monitoring boundary lines (*) for benefit and harm respectively. Two horizontal lines represents the conventional boundaries (†) for statistical significance. Triangular lines on the right side reflects the futility boundaries (‡). The number on the x-axis indicates RIS.

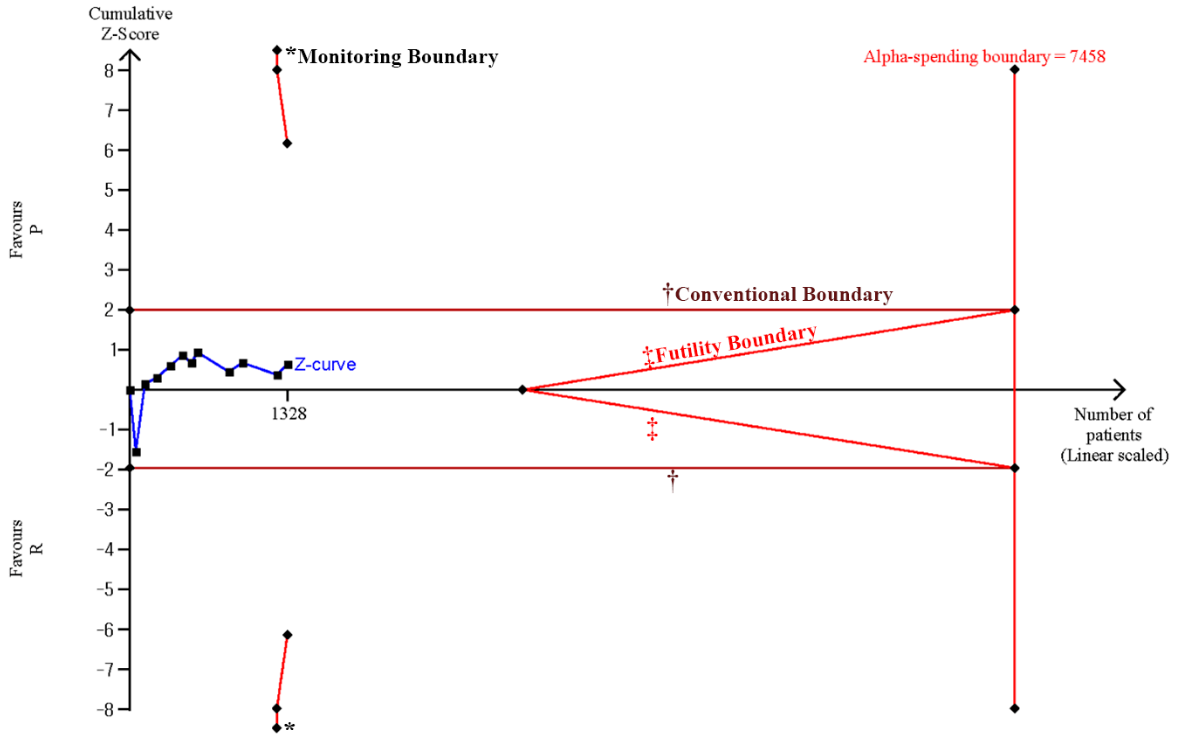

Supplementary Figure 4C. **The trial sequential analysis for the studies comparing the effect of palonosetron to that of ramosetron on the use of rescue anti-emetics**. The cumulative Z curve did not cross the conventional test boundary and stayed within the statistically insignificant zone. TSA indicated that only 1,488 of 7,458 patients of the required information size (RIS) accumulated. Uppermost and lowermost curves represent trial sequential monitoring boundary lines (*) for benefit and harm respectively. Two horizontal lines represents the conventional boundaries (†) for statistical significance. Triangular lines on the right side reflects the futility boundaries (‡). The number on the x-axis indicates RIS.

Group by Time	Study name	Risk ratio	Lower limit	Upper limit
Different	Yoon 2016	0.842	0.639	1.109
Different		0.842	0.639	1.109
Early	Chattopadhyay 2015	1.636	0.411	6.512
Early	Kim 2013	2.111	0.412	10.829
Early	Park 2013	0.250	0.056	1.119
Early	Piplai 2012	0.750	0.183	3.068
Early		0.886	0.424	1.849
Late	Kim 2015	1.000	0.000	6344.109
Late	Roh 2014	0.889	0.482	1.640
Late	Song 2017	1.460	0.850	2.508
Late	Ahluwalia 2015	1.000	0.064	15.588
Late	Vinvay 2017	1.000	0.219	4.564
Late		1.158	0.786	1.706
Overall		0.933	0.752	1.156

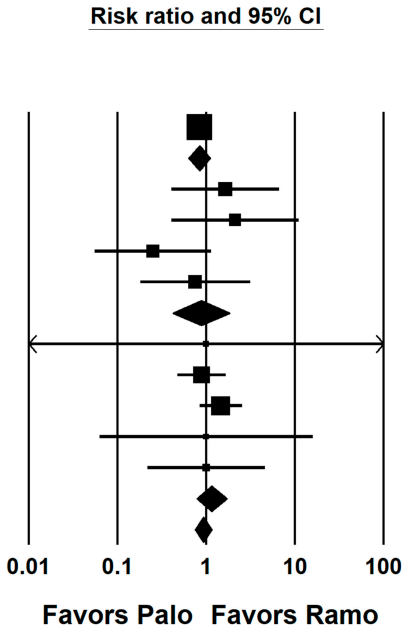

Supplementary Figure 5A. **Forest plot for studies comparing the effect of palonosetron to that of ramosetron on dizziness.** The combined results showed no evidence of a difference between palonosetron and ramosetron. For subgroup analysis, there was no evidence of differences between palonosetron and ramosetron according to the time of administration.

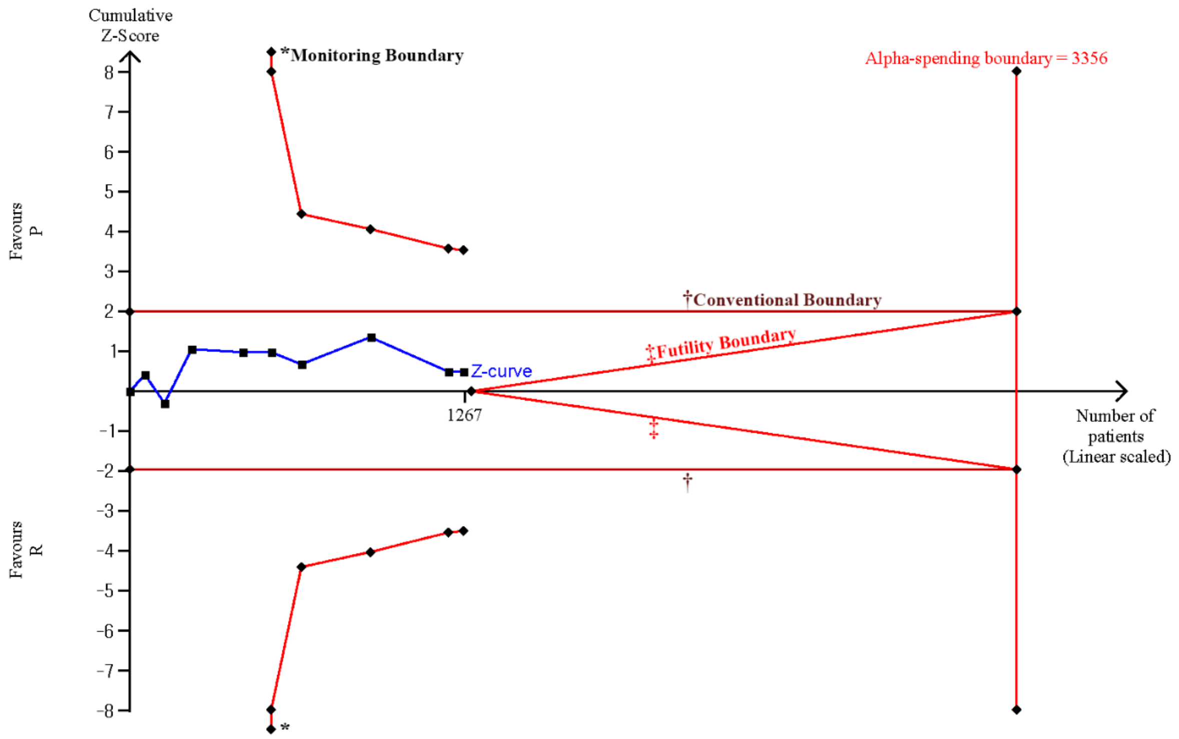

Supplementary Figure 5B. **The trial sequential analysis for the studies comparing the effect of palonosetron to that of ramosetron on dizziness.** The cumulative Z curve did not cross the conventional test boundary and stayed within the statistically insignificant zone. TSA indicated that only 1,355 of 3,356 patients of the required information size (RIS) accumulated. Uppermost and lowermost curves represent trial sequential monitoring boundary lines (*) for benefit and harm respectively. Two horizontal lines represents the conventional boundaries (†) for statistical significance. Triangular lines on the right side reflects the futility boundaries (‡). The number on the x-axis indicates RIS.

Group by Time	Study name	Risk ratio	Lower limit	Upper limit
Different	Bang 2017	0.333	0.014	7.860
Different	Yoon 2016	1.806	0.933	3.495
Different		1.682	0.881	3.211
Early	Chattopadhyay 2015	1.122	0.437	2.879
Early	Kim 2013	2.111	0.200	22.287
Early	Park 2013	1.000	0.433	2.309
Early	Piplai 2012	0.667	0.120	3.709
Early		1.042	0.589	1.843
Late	Roh 2014	1.000	0.473	2.116
Late	Song 2017	1.541	0.770	3.085
Late	Ahluwalia 2015	1.250	0.354	4.409
Late	Vinvay 2017	0.400	0.084	1.903
Late		1.145	0.729	1.799
Overall		1.217	0.892	1.660

Risk ratio and 95% CI

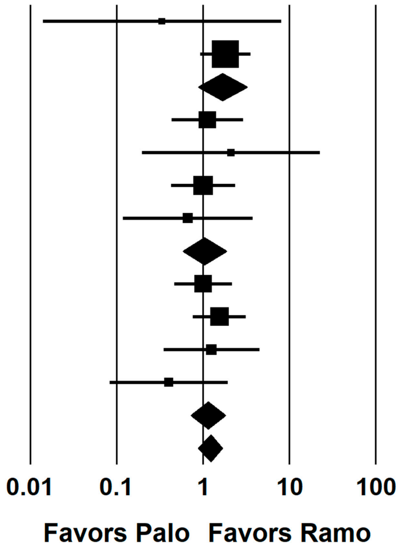

Supplementary Figure 5C. Forest plot for studies comparing the effect of palonosetron to that of ramosetron on **headache**. The combined results showed no evidence of a difference between palonosetron and ramosetron. For subgroup analysis, there was no evidence of differences between palonosetron and ramosetron according to the time of administration.

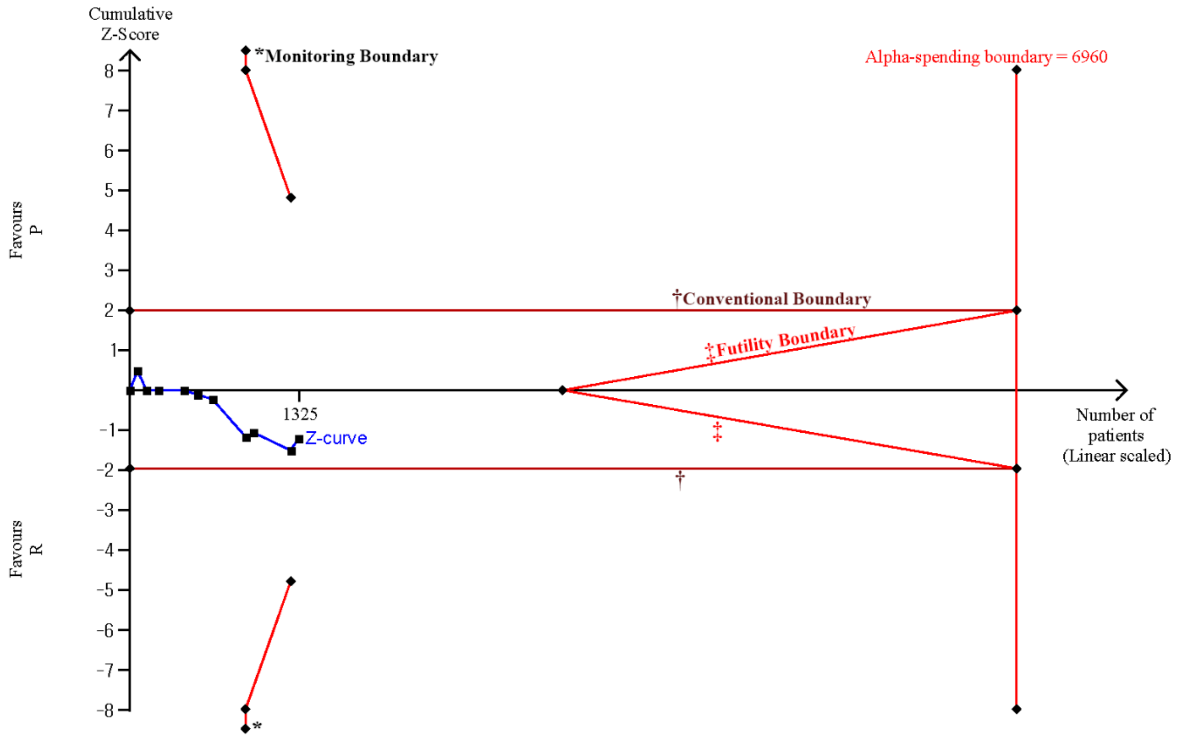

Supplementary Figure 5D. The trial sequential analysis for the studies comparing the effect of palonosetron to that of ramosetron on headache. The cumulative Z curve did not cross the conventional test boundary and stayed within the statistically insignificant zone. TSA indicated that only 1,413 of 6,960 patients of the required information size (RIS) accumulated. Uppermost and lowermost curves represent trial sequential monitoring boundary lines (*) for benefit and harm respectively. Two horizontal lines represents the conventional boundaries (†) for statistical significance. Triangular lines on the right side reflects the futility boundaries (‡). The number on the x-axis indicates RIS.
